# Enhancement of Ni-NiO-CeO_2_ Interaction on Ni–CeO_2_/Al_2_O_3_-MgO Catalyst by Ammonia Vapor Diffusion Impregnation for CO_2_ Reforming of CH_4_

**DOI:** 10.3390/molecules29122803

**Published:** 2024-06-12

**Authors:** Sabaithip Tungkamani, Saowaluk Intarasiri, Wassachol Sumarasingha, Tanakorn Ratana, Monrudee Phongaksorn

**Affiliations:** 1 Department of Industrial Chemistry, Faculty of Applied Science, King Mongkut’s University of Technology North Bangkok, Bangkok 10800, Thailand; sabaithip.t@sci.kmutnb.ac.th (S.T.); w.sumarasingha@gmail.com (W.S.); tanakorn.r@sci.kmutnb.ac.th (T.R.); 2 Research and Development Center for Chemical Engineering Unit Operation and Catalyst Design (RCC), King Mongkut’s University of Technology North Bangkok, Bangkok 10800, Thailand; 3 Faculty of Science, Energy and Environment, King Mongkut’s University of Technology North Bangkok, Rayong 21120, Thailand; saowaluk.i@sciee.kmutnb.ac.th

**Keywords:** CO_2_ reforming of methane, Ni-CeO_2_ catalyst, Ni-NiO-CeO_2_ interaction, surface hydroxyl, coke deposition

## Abstract

Ni-based catalysts have been widely used for the CO_2_ reforming of methane (CRM) process, but deactivation is their main problem. This study created an alternative electronic Ni-NiO-CeO_2_ interaction on the surface of 5 wt% Ni-5 wt% CeO_2_/Al_2_O_3_-MgO (5Ni5Ce(xh)/MA) catalysts to enhance catalytic potential simultaneously with coke resistance for the CRM process. The Ni-NiO-CeO_2_ network was developed on Al_2_O_3_-MgO through layered double hydroxide synthesis via our ammonia vapor diffusion impregnation method. The physical properties of the fresh catalysts were analyzed employing FESEM, N_2_ physisorption, and XRD. The chemical properties on the catalyst surface were analyzed employing H_2_-TPR, XPS, H_2_-TPD, CO_2_-TPD, and O_2_-TPD. The CRM performances of reduced catalysts were evaluated at 600 °C under ambient pressure. Carbon deposits on spent catalysts were determined quantitatively and qualitatively by TPO, FESEM, and XRD. Compared to 5 wt% Ni-5 wt% CeO_2_/Al_2_O_3_-MgO prepared by the traditional impregnation method, the electronic interaction of the Ni-NiO-CeO_2_ network with the Al_2_O_3_-MgO support was constructed along the time of ammonia diffusion treatment. The electronic interaction in the Ni-NiO-CeO_2_ nanostructure of the treated catalyst develops surface hydroxyl sites with an efficient pathway of OH* and O* transfer that improves catalytic activities and coke oxidation.

## 1. Introduction

The unprecedented climate change mainly caused by greenhouse gases is becoming critical. Up to the present day, alternative sources of energy have been generated and developed to decrease greenhouse gas emissions [1,2]. The concentration of greenhouse gases is still at an all-time high. Carbon dioxide reforming of methane (CRM) (Equation (1)) is a significant catalytic process that commercially converts two key greenhouse gases (methane (CH_4_) and carbon dioxide (CO_2_)) into syngas, a gas mixture composed primarily of hydrogen (H_2_) and carbon monoxide (CO) [3,4,5]. The syngas produced with an approximately equal H_2_/CO ratio from the CRM process can be effectively utilized as a precursor for chemical industry production and for green fuel synthesis. In recent decades, Ni-based formulations have been considered as practical CRM catalysts because of their substantial activity with economic cost. However, fast and continuous deactivation is a critical issue for traditional Ni-based catalysts. During the reaction, Ni-based-catalyst-supported metal oxide can be impeded by metal sintering and coke deposition [6,7,8,9]. Over the catalyst surface, metal sintering is caused by a high operating temperature, while coke is formed and grown via side reactions, methane cracking (Equation (2)), and the Boudouard reaction (Equation (3)). Moreover, the side reaction that consumes H_2_ and causes the low H_2_/CO ratio in the syngas product is the reverse water–gas shift (RWGS, Equation (4)) [10,11].

CH_4_ + CO_2_ → 2H_2_ + 2CO     △H^o^_298 K_ = +247 kJ/mol (1)

CH_4_ → 2H_2_ + C     △H^o^_298 K_ = +75 kJ/mol (2)

2CO → CO_2_ + C     △H^o^_298 K_ = +173 kJ/mol (3)

H_2_ + CO_2_ → H_2_O + CO     △H^o^_298 K_ = +41 kJ/mol (4)

Several studies revealed how CeO_2_ improves the catalyst activity and stability of Ni-based catalysts. When CeO_2_ plays a vital role as a support for Ni-based catalysts, Ni sintering and coke deposition can be simultaneously resisted through the Ni-CeO_2_ interaction and oxygen storage properties of CeO_2_. Nonetheless, CeO_2_ has a low surface area compared to other available supports (Al_2_O_3_, MgO-Al_2_O_3_, SBA-15) [12,13,14]. As a promoter, the addition of CeO_2_ to a Ni/Al_2_O_3_ catalyst forms Ce_1−X_Ni_X_O_2_ and CeAlO_3_ phases on the catalyst surface. Thus, the oxygen transfer and reducibility of the promoted catalyst can be improved to raise its catalytic activity and carbon resistance [15,16,17,18].

Our group has developed a novel ammonia vapor diffusion impregnation method that allows the size of the hierarchical nanostructures of the modified catalyst surface to be controlled by the ammonia vapor diffusion time. Previously, the hierarchical structure and basicity of the layered nanosheets on 10 wt% Ni/MgO-Al_2_O_3_ prepared by ammonia vapor diffusion impregnation for 20 h exhibited that the hierarchical nanostructures on the surface can enhance CO_2_ adsorption–dissociation and prevent coke formation for the CRM process [19]. A 10 wt% Ni-1 wt% ZrO_2_/Al_2_O_3_ catalyst prepared by a similar method with an ammonia diffusion time of 6 h demonstrated an improvement in the steam activation–dissociation and coke resistance for the combined steam and CO_2_ reforming of methane (CSCRM) process operated under low-temperature and low-steam conditions. This can be attributed to the very dispersive nanosheets and redox properties of the ZrO_2_ promoter that produces the number of the OH* and O* species on the surface [20]. Consequently, it would be interesting to develop the heterogeneity of Ni-CeO_2_ networks on catalyst surface through time-controlled ammonia diffusion treatment to investigate its effect on properties, CRM performance, and deactivation.

Herein, this present work prepared a 5 wt% Ni-5 wt% CeO_2_-supported MgO-Al_2_O_3_ catalyst by the ammonia vapor diffusion impregnation method (5Ni5Ce(xh)/MA). During the preparation, a nickel–cerium network nanostructure was initiated and grown during the ammonia diffusion treatment. Accordingly, two treatment times (6 h and 12 h) were applied to study the electronic effects of size and different network nanostructure morphologies on the catalyst surface. Thus, the physical properties were obtained via field emission scanning electron microscopy (FESEM), N_2_ physisorption analysis, and X-ray diffraction (XRD) analysis. The chemical insights into the surface interactions of the nanostructure on 5Ni5Ce(xh)/MA were evaluated by employing X-ray photoelectron spectroscopy (XPS), temperature-programmed reduction in H_2_ (H_2_-TPR), temperature-programmed desorption of H_2_ (H_2_-TPD), temperature-programmed desorption of O_2_ (O_2_-TPD), and temperature-programmed desorption of CO_2_ (CO_2_-TPD). The CRM catalytic performance was demonstrated in a tubular reactor at 600 °C for 16 h under atmospheric pressure. After the reaction, the carbon deposition was assessed using temperature-programmed oxidation (TPO), FESEM, and XRD. The above-mentioned effects on catalytic characteristics were elaborated and compared to the 5 wt% Ni-5 wt% CeO_2_-supported MgO-Al_2_O_3_ prepared by the traditional impregnation method (5Ni5Ce/MA).

## 2. Results and Discussion

### 2.1. Physical Properties

**Figure 1:**
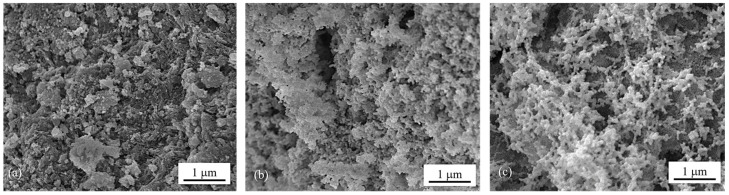
FESEM micrographs of calcined (**a**) 5Ni5Ce/MA, (**b**) 5Ni5Ce(6 h)/MA, and (**c**) 5Ni5Ce(20 h)/MA catalysts.

The surface morphologies of the calcined catalysts were investigated by means of FESEM images (Figure 1a–c) which show the changes in surface structure caused by ammonia vapor treatment. Compared to the morphology of 5Ni5Ce/MA (Figure 1a), the network of the metal oxide nanoparticles is generated over the surface of 5Ni5Ce(6 h)/MA (Figure 1b). This network of metal oxide nanoparticles is clearly visible on 5Ni5Ce(20 h)/MA (Figure 1c), implying the growth of the NiO-CeO_2_ interaction throughout the ammonia vapor diffusion. This can be explained by layered double hydroxide (LDH) synthesis via ammonia vapor diffusion impregnation [19,20,21]. When ammonia vapor is dissolved into the impregnated nickel–cerium nitrate solution on the support, a limited amount of hydroxide ions are produced, conducting the slow growth of the layered double hydroxide ([Ni_1−-x_Ce_x_(OH)_2_]^z+^(NO_3_)_z_·nH_2_O) throughout the diffusion time [22,23,24]. In this complex, metal ions are connected based on the inorganic layer as a multicomponent nanostructure. After calcination, the layer becomes a mixed-metal-oxide nanostructure.

**Figure 2:**
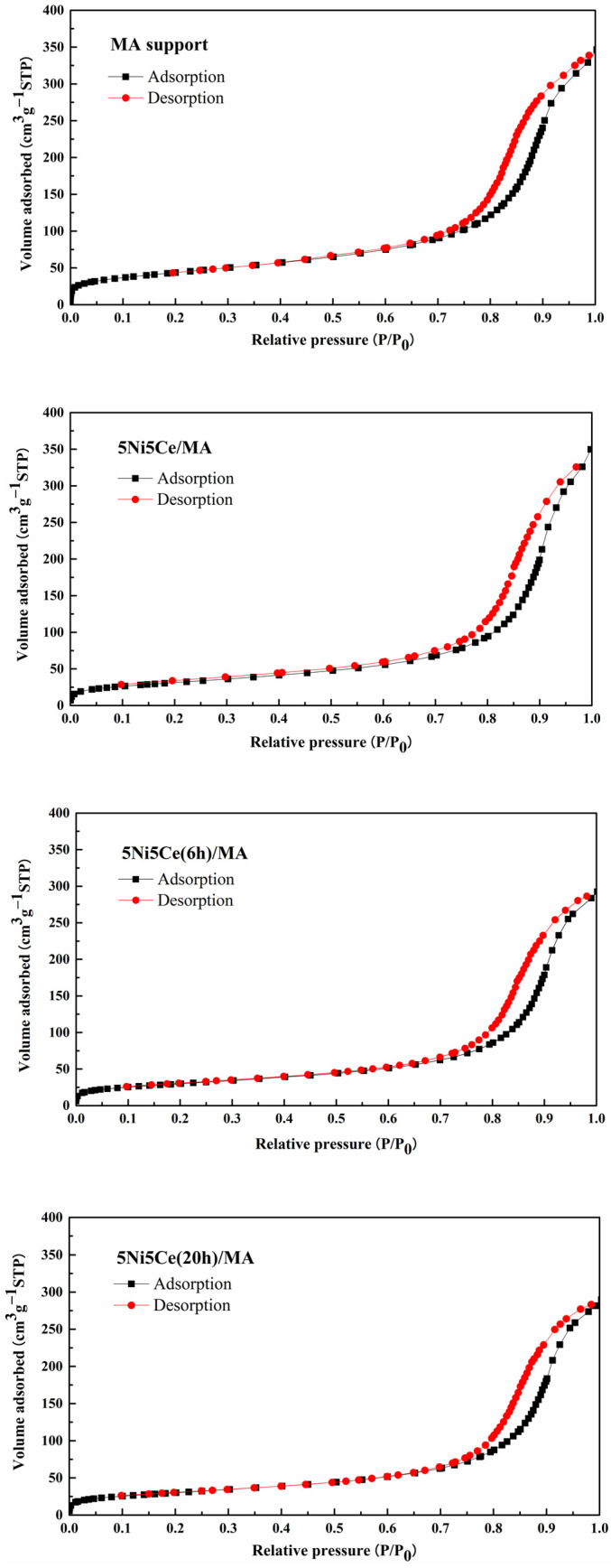
N_2_ adsorption–desorption isotherms of support and all calcined catalysts.

**Figure 3:**
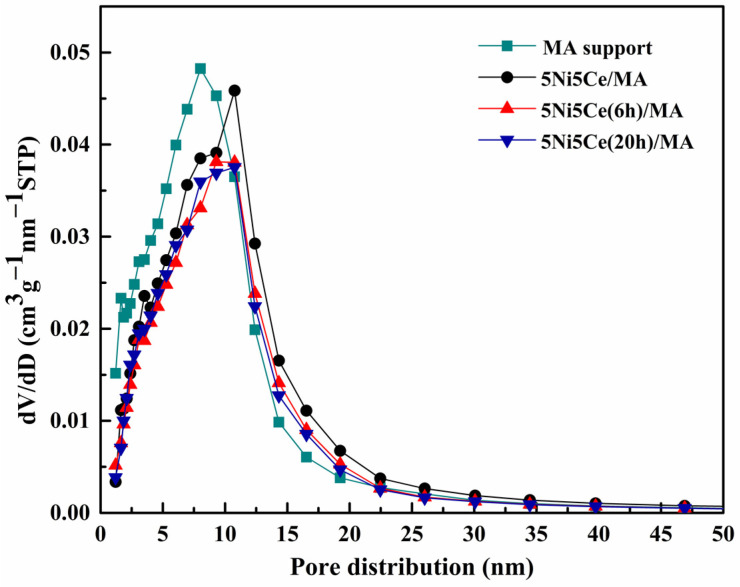
BJH pore size distributions of support and all calcined catalysts.

The textural properties of all samples were examined using N_2_ adsorption–desorption measurements. The isotherm patterns of all samples (Figure 2) exhibit a type IV(a) isotherm feature of mesoporous materials with the combination of H2(b) and H3 hysteresis loops (IUPAC), correlating to the connection of the bottleneck-shaped and plate-shaped pores in the relative pressure range (P/P_0_) of 0.60–1.00 [25,26,27,28]. Using the BJH method, the pore size distribution curves of all samples are established in Figure 3. Table 1 reports the average pore size diameter estimated from the BJH method and the surface area with the total pore volume calculated from the BET equation. The support and catalyst samples show an average pore size diameter smaller than 25.0 nm. The surface of the MA support has an area of 151 m^2^ g^−1^ with an average pore size diameter of 8.0 nm and a total pore volume of 0.52 cm^3^ g^−1^. When 5 wt% Ni and 5 wt% CeO_2_ were loaded onto the MA support (5Ni5Ce/MA), the surface area decreased to 108 m^2^ g^−1^ with a total pore volume of 0.51 cm^3^ g^−1^, whereas the average pore size diameter increased to 10.8 nm. These results can be explained by the blockage of smaller pores of the MA support by large nanoparticles [29,30]. Considering the ammonia vapor diffusion impregnation of 5 wt% Ni and 5 wt% CeO_2_ for 6 h (5Ni5Ce(6 h)/MA) and 20 h (5Ni5Ce(20 h)/MA), the surface area and total pore volumes of these two catalysts are similar (101 m^2^ g^−1^ and 0.43–0.44 cm^3^ g^−1^, respectively). These values of the textural properties are lower than 5Ni5Ce/MA due to the stronger blocking effect caused by the growth of the mixed-oxide nanoparticles.

**Figure 4:**
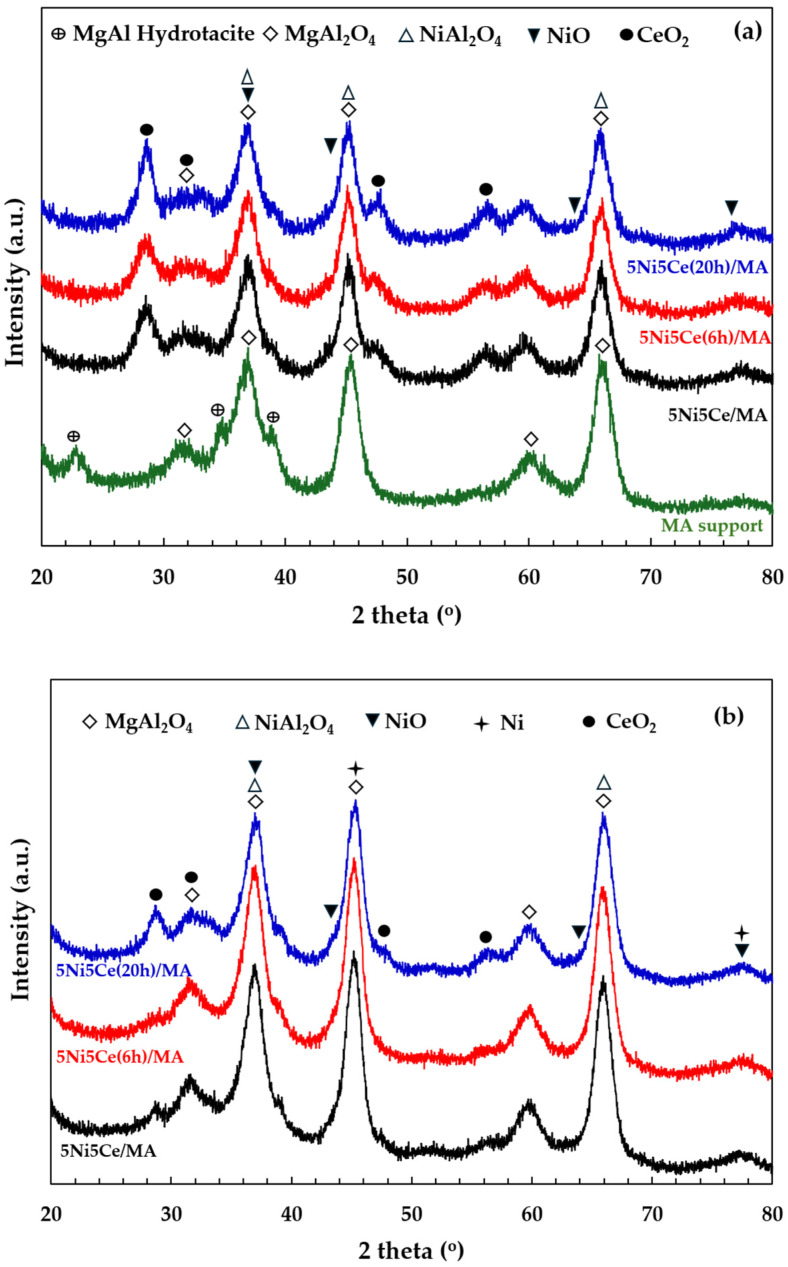
The X-ray diffraction patterns of (**a**) MA support and calcined catalysts, and (**b**) reduced catalysts.

The X-ray diffraction patterns of the MA support, calcined catalysts, and reduced catalysts are presented in Figure 4. According to Figure 4a, the diffractogram of the MA support represents the peak patterns of the MgAl hydrotalcite phase (JCPDS No. 022-0700) and the MgAl_2_O_4_ spinel structure (JCPDS No. 01-084-0377) [31,32,33,34]. After calcination in the catalyst preparation, the MgAl hydrotalcite phase is completely transformed into the MgAl_2_O_4_ spinel phase, as the pattern of MgAl hydrotalcite cannot be observed in the diffractograms of the calcined catalysts (Figure 4a). When the 5 wt.% Ni and 5 wt.% CeO_2_ were impregnated onto the MA support, the pattern of the spinel phase could also be attributed to the occurrence of NiAl_2_O_4_ (JCPDS No. 10-0339) on all of the calcined catalysts. This pattern overlaps with the diffraction peaks of MgAl_2_O_4_ spinel and NiO crystalline phase (JCPDS No. 47-1049). Likewise, nickel oxide phases and the nickel metal phase (JCPDS No. 04-0850) are seldom identified in the diffractograms of the reduced catalysts (Figure 4b) due to the overlapping peaks in the patterns of various nickel crystalline systems [35,36,37]. Thus, the nickel species on the reduced catalysts were investigated via XPS characterization. Differently, the peak patterns of CeO_2_ (JCPDS No. 43-1002) were detected in the diffractograms of all the calcined catalysts and reduced catalysts [38,39,40,41]. For a quantitative comparison, the CeO_2_ crystallite sizes on the calcined catalysts (Table 1) were determined via Scherrer’s equation at 2 theta = 28°. Compared to the calcined 5Ni5Ce/MA, the average CeO_2_ crystallite size slightly decreased when the catalyst was treated in ammonia vapor for 6 h, indicating a greater distribution of the NiO-CeO_2_ nanostructure. Contrarily, the crystallite size of CeO_2_ on the surface increased when the catalyst was treated in ammonia vapor for 20 h, implying the growth of a mixed-metal-oxide nanostructure during the time of treatment. Although the crystallite sizes of CeO_2_ on the reduced catalysts were smaller than those of the calcined catalysts due to the partial reduction in CeO_2_, the same CeO_2_ crystallite size trend was observed on the reduced catalysts.

### 2.2. Chemical Properties

**Figure 5:**
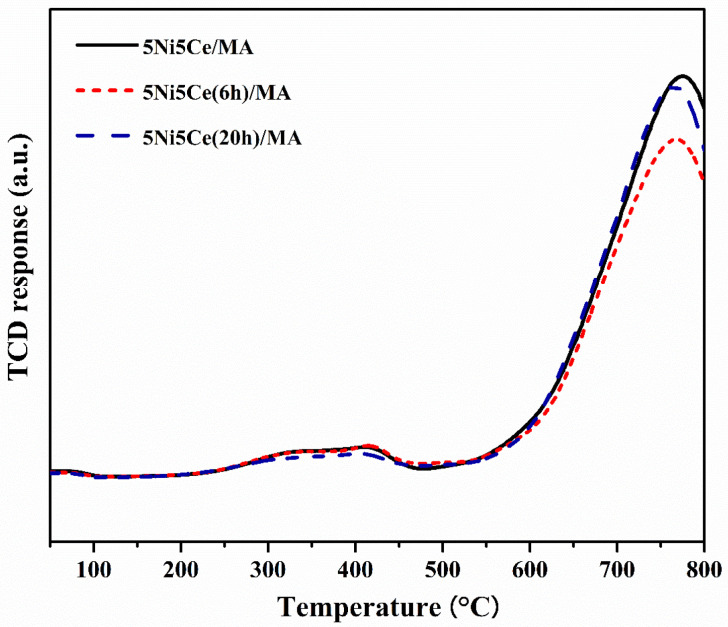
H_2_-TPR profiles of all reduced catalysts.

**Figure 6:**
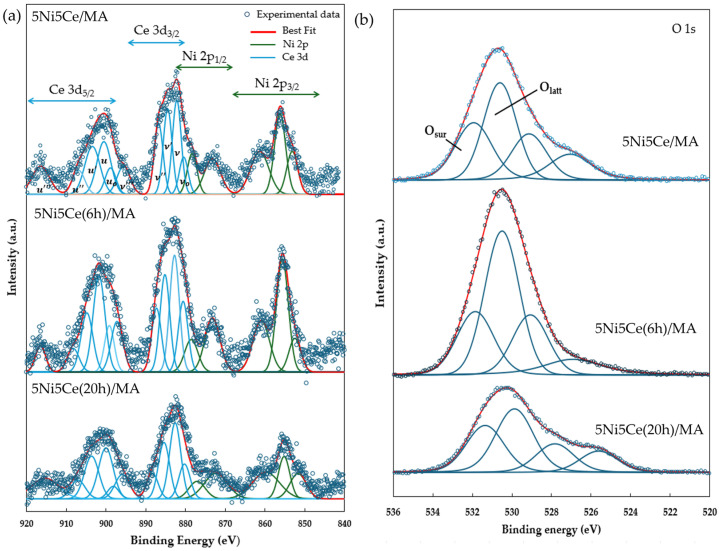
XPS core level spectra of reduced catalysts in (**a**) Ni 2p + Ce 3d energy region and (**b**) O 1s energy region.

The reduction profiles of the oxide species (Figure 5) were carried out by the H_2_ temperature program reduction (H_2_-TPR) to determine the reducibility and the interaction among the components of the calcined catalysts. The H_2_-TPR profiles of all the calcined catalysts demonstrate two temperature reduction ranges. The reductions at lower temperature ranging from 250 °C to 450 °C correspond to the NiO species poorly interacting with other components and the partial reduction in CeO_2_. The reduction peaks at temperatures higher than 500 °C are assigned to the moderate-to-strong interaction of NiO-CeO_2_ and NiO-CeO_2_-MA and the formation of spinel oxides such as NiAl_2_O_4_ due to the strong metal–support interaction (SMSI) effect [42,43,44,45,46,47]. The XPS spectra of Ni 2p + Ce 3d (Figure 6a) and O 1s (Figure 6b) were obtained to investigate the chemical states of Ni and Ce on the surface of all ex situ reduced catalysts. In Figure 6a, the Ni 2p core level spectra show the presence of metallic Ni (Ni^0^) at a binding energy (BE) of 851.7–852.9 eV, and Ni^2+^ at a BE of 855.2–855.9 eV. The Ce 3d core level spectra show the co-existence of Ce^4+^ (blue peaks = v, v″, v‴, u, u″, and u‴) and Ce^3+^ (blue peaks = v_0_, v′, u_0_, and u′) [48,49,50,51]. The O 1s peak (Figure 6b) was deconvoluted to identify the oxygen species of the reduced catalysts. The peaks of lattice oxygen (O_latt_) in NiO, CeO_2_, NiAl_2_O_4_, and the MA support was found at a BE of about 530 eV. The peaks of surface oxygen (O_sur_) based on oxygen vacancies and hydroxyl were observed at higher BE values (531–532 eV) [52,53]. The relative concentration of metallic nickel Ni ([Ni^0^]/[Ni^0^] + [Ni^2+^]), the relative concentration of Ce^3+^ ([Ce^3+^]/[Ce^4+^] + [Ce^3+^]), and the O_sur_/O_latt_ ratio were then evaluated from their peak areas (Table 2).

According to the H_2_-TPR profiles and XPS results, the maximum hydrogen consumption with the reduction curve at the highest temperature and the maximum relative concentrations of Ni^0^ and Ce^3+^ with the highest O_sur_/O_latt_ ratio were obtained from the untreated 5Ni5Ce/MA catalyst. These results indicate the greatest reductions in nickel oxide (NiO or NiAl_2_O_4_ → Ni^0^) and CeO_2_ (CeO_2_ (Ce^4+^)→ Ce_2_O_3_ (Ce^3+^), generating oxygen vacancies) with the presence of spinel phases due to the SMSI effect on the surface of the untreated 5Ni5Ce/MA catalyst. The reduction peak slightly shifts to a lower temperature with the minimum hydrogen consumption on 5Ni5Ce(6 h)/MA. The smallest amount of nickel oxide reduction was confirmed by the lowest Ni^0^ fraction with the lower partial CeO_2_ reduction (XPS result). This is owing to the high dispersion of the initiated NiO-CeO_2_ nanoparticles on the MA support during the short treatment process. Therefore, the SMSI effect still presents, and the tiny nanoparticles cause some nickel oxide particles to merge completely into the MA support underneath the surface with the NiO-CeO_2_ interaction. Consequently, these deduct the reductions in nickel oxide as well as in CeO_2_ and show a greater O_latt_ portion on the surface. When treated by ammonia vapor a for longer time (5Ni5Ce(20 h)/MA catalyst), the NiO-CeO_2_ network (SEM image) constructed on the surface increases nickel oxide in the mixed-oxide nanoparticles and decreases the isolated CeO_2_. Compared to 5Ni5Ce(6 h)/MA, hydrogen consumption at a high temperature range and the relative concentration of Ni^0^ increases while the relative concentration of Ce^3+^ decreases on 5Ni5Ce(20 h)/MA. Considering the quantity of Ni^0^, the hydrogen consumption profiles of H_2_-TPR of 5Ni5Ce/MA and 5Ni5Ce(20 h)/MA are almost similar, while the Ni^0^ relative concentrations are different. This indicates the number of Ni^2+^ in other forms. For the treated catalyst with which the surface was constructed in the LDH formation for a long time, the NiO-CeO_2_ mixed oxide increased the Ni^2+^ on the surface, forming Ni(OH)_2_ and NiO. Moreover, only NiO at the uppermost level can be reduced. Therefore, 5Ni5Ce(20 h)/MA shows a higher O_sur_/O_latt_ ratio than that of 5Ni5Ce(6 h)/MA.

**Figure 7:**
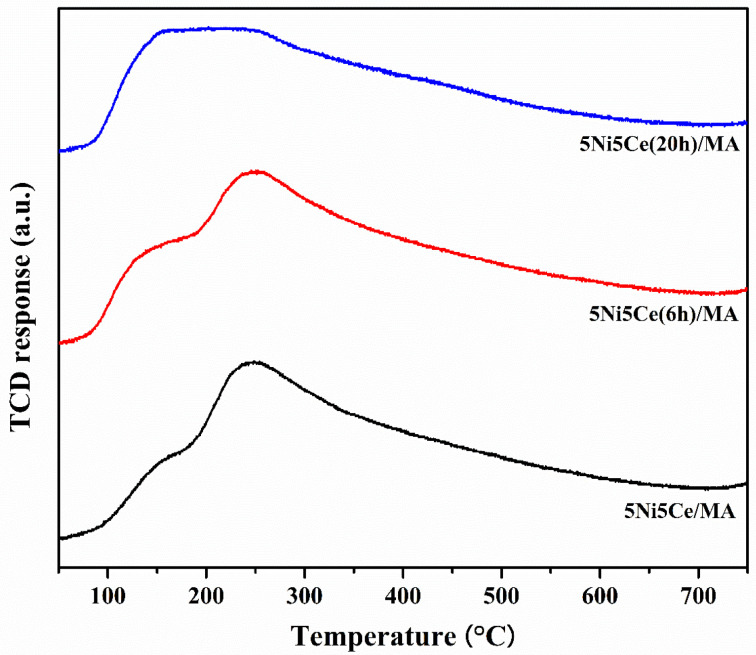
H_2_-TPD profiles of all reduced catalysts.

The metal particle size distributions, including the metal surface layers of the reduced catalysts, were determined via H_2_-TPD measurement. Two desorption temperature ranges were observed in the profiles (Figure 7), suggesting a weak hydrogen chemisorption on the Ni metal at the top layer (temperature lower than 180 °C) and the medium–strong hydrogen chemisorption on the Ni metal interacting with the support, contributing to the hydrogen spillover onto the catalyst support (temperatures higher than 180 °C) [54,55,56]. The amount of hydrogen desorption was determined as reported in Table 3. The H_2_-TPD profiles of the reduced catalysts represent different arrangements of Ni metal active sites on the surface. The largest hydrogen desorption at a high temperature range and the smallest hydrogen desorption at a low temperature range were obtained from the untreated catalyst, implying that most Ni metal active sites interacted with the support. The 5Ni5Ce(6 h)/MA catalyst possesses the largest amount of H_2_ desorption, representing the highest Ni dispersion. According to the H_2_-TPD profiles of the treated catalysts, the hydrogen desorption at high temperature ranges trends to decrease, while the hydrogen desorption at low temperature ranges trends to increase when the duration of the ammonia diffusion treatment increases. These results suggest the raising of the Ni metal active sites on the uppermost surface layer due to the growth of the NiO-CeO_2_ layer on the surface, which reduces the interaction of active sites with the support.

**Figure 8:**
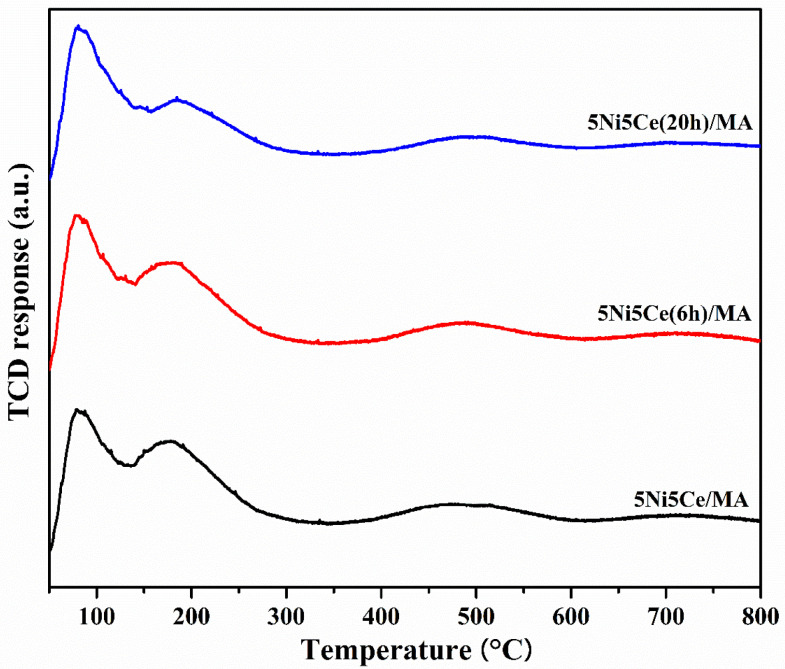
CO_2_-TPD profiles of all reduced catalysts.

The amount and strength of the basic sites on the reduced catalysts related to the catalytic activity of CO_2_ were identified by CO_2_-TPD (Figure 8). The strength of the basic sites can be distinguished by the temperature corresponding to the desorption of CO_2_. The basic sites of reduced 5Ni5Ce/MA, 5Ni5Ce(6 h)/MA, and 5Ni5Ce(20 h)/MA catalysts demonstrate three temperature ranges of CO_2_ desorption. The weak basic sites in the low temperature range of 50–150 °C can be ascribed to Brønsted OH^−^ groups on the surface, the moderate basic sites (150–400 °C) are attributed to metal–oxygen pairs (Lewis basic site), and the strong basic sites at temperatures higher than 400 °C are assigned to the surface of low coordinated O^2−^ anions [57,58,59]. Among all the strengths of the basic sites, the Brønsted OH^–^ and Lewis basic sites are clearly observed on all reduced catalysts. Table 4 reports the deconvoluted quantities of CO_2_ desorption from all reduced catalysts. Accordingly, the treated catalysts present a greater number of Brønsted OH^−^ sites than the untreated 5Ni5Ce/MA catalyst, and this type of basic site tends to increase with the duration of treatment. This can be explained by the effect of surface modification by treatment in ammonia vapor diffusion, which composes the surface through the LDH structure and involves the rehydration property (memory effect) after calcination.

**Figure 9:**
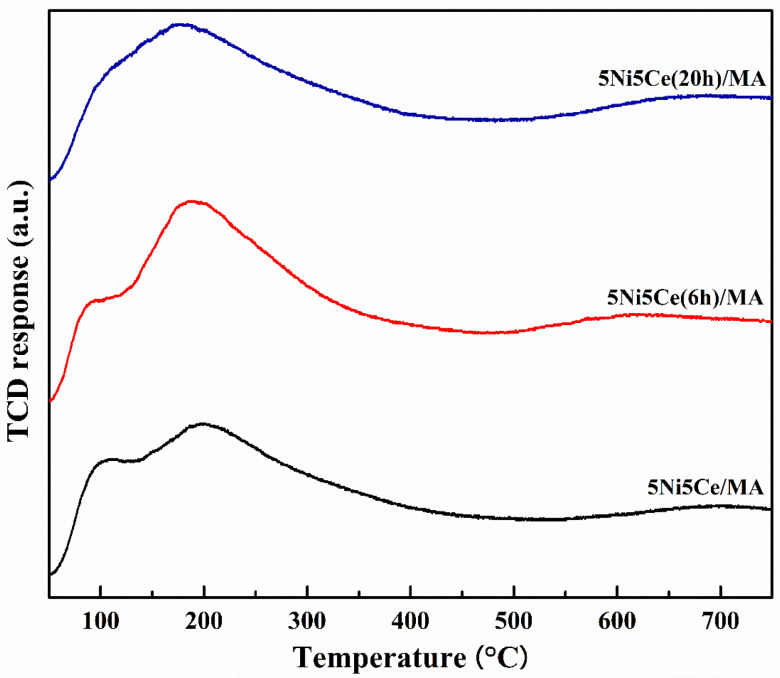
O_2_-TPD profiles of all reduced catalysts.

The oxygen mobility in all reduced catalysts was further evaluated by O_2_-TPD measurement (Figure 9) at a temperature range from 50 °C up to 800 °C. The oxygen transfer on the catalyst can be predicted via the O_2_-TPD profile. The oxygen desorption peaks at low temperature are assigned to surface oxygen, including physiosorbed oxygen molecules and oxygen from oxygen vacancies (resulting from the redox property of Ce^4+^/Ce^3+^). At the higher temperature range, the desorption peaks are attributed to lattice oxygen [16,60,61,62,63,64,65]. The oxygen desorption quantities according to the various types of oxygen mobility were calculated (Table 5). The O_2_-TPD results may be relative to the XPS analysis. The 5Ni5Ce/MA catalyst shows two desorption peaks at different ranges of temperature. Compared to 5Ni5Ce/MA, the catalyst treated in the ammonia vapor for 6 h (5Ni5Ce(6 h)/MA catalyst) presents less surface oxygen and more lattice oxygen due to the good dispersion of small NiO-CeO_2_ nanoparticles initiated on the surface. For the comparison of treated catalysts (6 h and 20 h), surface oxygen increases, lattice oxygen decreases, and the peaks of surface and lattice oxygen merge smoothly in the O_2_-TPD profile of 5Ni5Ce(20 h)/MA. These results indicate the connection of the surface and lattice oxygen through the electronic interaction of Ni-NiO-CeO_2_ produced by 20 h ammonia vapor treatment.

### 2.3. Catalytic Performance and Coke Resistance

**Figure 10:**
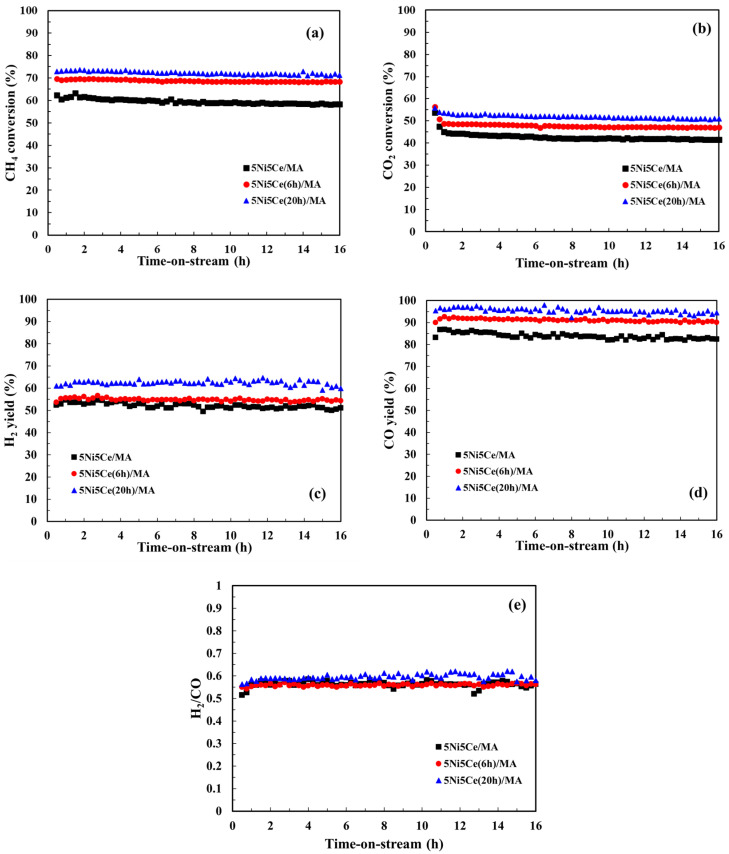
(**a**) CH_4_ conversion, (**b**) CO_2_ conversion, (**c**) H_2_ yield, (**d**) CO yield, and (**e**) H_2_/CO ratio for the CO_2_ reforming of methane (CRM) of all catalysts. Reaction conditions: 600 °C and 1 atm for 16 h.

The CMR catalytic behaviors of the 5Ni5Ce/MA and treated 5Ni5Ce(xh)/MA catalysts were investigated at 600 °C under atmospheric pressure for 16 h. The performance was evaluated by means of CH_4_ conversion, CO_2_ conversion, and the H_2_/CO ratio of the syngas product (Figure 10). The 5Ni5Ce/MA catalyst presents an average CH_4_ conversion of 59.4%, an average CO_2_ conversion of 43.5%, and a H_2_/CO ratio of 0.58 (H_2_ yield = 51.8% and CO yield = 82.7%). Compared to 5Ni5Ce/MA, the 5Ni5Ce(6h)/MA catalyst reflects the relative higher activity toward CH_4_ conversion (68.8%) and CO_2_ conversion (47.9%) with similar H_2_/CO ratio of 0.56 (H_2_ yield = 54.8% and CO yield = 90.9%). The highest CH_4_ conversion of 72.2%, the highest CO_2_ conversion of 52.1%, and a H_2_/CO ratio of 0.59 (H_2_ yield = 62.3% and CO yield = 95.3%) were obtained from the 5Ni5Ce(20h)/MA catalyst. The enhancement of reactant activities as well as product yields on 5Ni5Ce(20h)/MA can be attributed to the number of active sites in the Ni-NiO-CeO_2_ network. As observed in CO_2_-TPD, these Ni-NiO-CeO_2_ nanostructures develop their electronic interactions with the nanostructure over the course of treatment. The surface hydroxide on the structure increases the turnover frequency of CO_2_ on the Ni-NiO-CeO_2_ network, agreeing with the CO_2_-TPD measurement. These surface hydroxyl sites allow Ni metal sites to be active toward CH_4_. For the CO yield values in which CH_4_ and CO_2_ consumptions were considered, the treated catalysts had smaller amounts of carbon deposition than the untreated catalyst. The coke deposition analysis is discussed in the following part. Moreover, the highest H_2_ yield (62.3%), the highest CO yield (95.3%) and the slightly higher H_2_/CO ratio (0.59) observed on the 5Ni5Ce(20 h)/MA catalyst imply a reduced RWGS side reaction effect. This is assigned to the rehydration property promoting H_2_O adsorption–dissociation [20].

**Figure 11:**
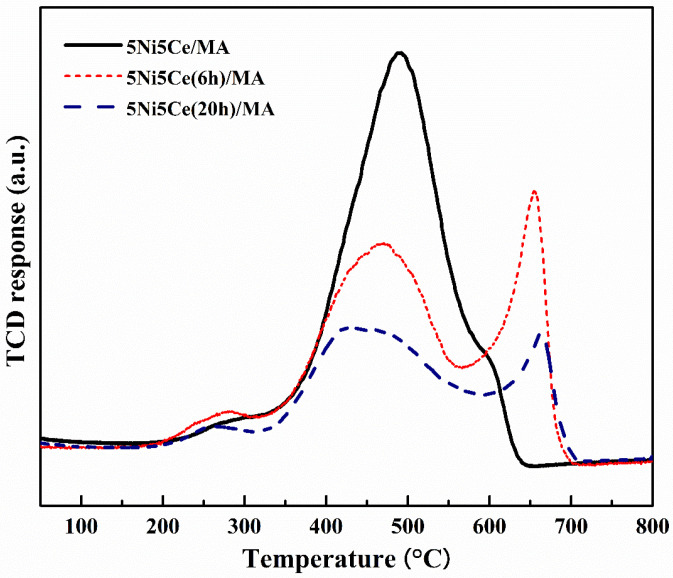
TPO profile of spent catalysts after CRM reaction.

**Figure 12:**
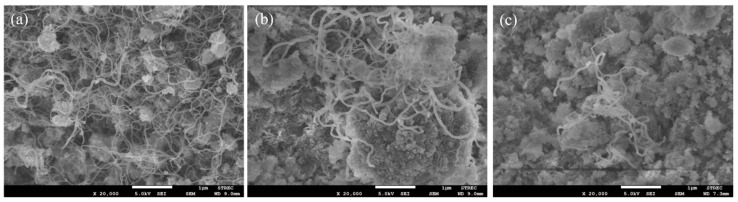
FESEM micrographs of spent (**a**) 5Ni5Ce/MA, (**b**) 5Ni5Ce(6 h)/MA, and (**c**) 5Ni5Ce(20 h)/MA catalysts.

**Figure 13:**
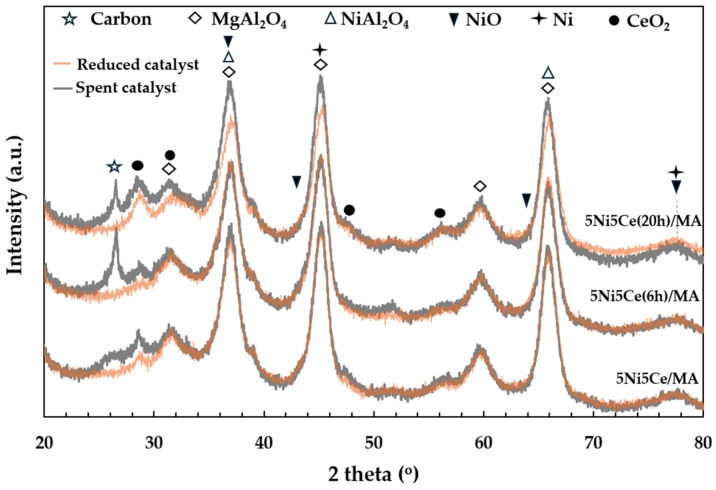
The X-ray diffraction patterns of the reduced catalysts and spent catalysts.

The carbon deposition on each spent catalyst after the CRM test was analyzed by TPO measurement (Figure 11), FESEM (Figure 12), and XRD (Figure 13). The oxygen consumption curves representing the carbon oxidation profiles of all samples are depicted in Figure 10, and the amount of oxygen consumptions are reported in Table 6. The oxidation peaks in the low-to-medium-temperature (<500 °C) and high-temperature (>500 °C) ranges can be assigned to the combustion of amorphous carbon and graphitic carbon collaborating with filamentous carbon, respectively [16,66,67,68,69,70]. The greatest amount of total oxygen consumption found on the spent 5Ni5Ce/MA suggests the largest amount of coke deposition. It decreased by 30% on the spent 5Ni5Ce(6 h)/MA and decreased by half on the spent 5Ni5Ce(20 h)/MA. Although a large amount of filamentous carbon was spread out over the surface of 5Ni5Ce/MA (Figure 12a–c), this carbon filament on the spent 5Ni5Ce/MA was oxidized easier than the treated catalysts due to the lower oxidation temperature. The XRD diffractograms of the reduced and spent catalysts establish the phase maintained during the reaction with the deposition of graphitic carbon (Figure 13). The XRD diffractograms of the spent catalysts display the peak patterns of graphitic carbon (JCPDS No. 41-1487) only on the treated catalysts [16,71]. This evidence reflects the change in the electronic effect on the nickel–ceria catalyst caused by the ammonia diffusion impregnation method. The electronic interaction of NiO-CeO_2_ developed through LDH synthesis generates and grows surface hydroxyl with the oxygen transfer pathway of the Ni-NiO-CeO_2_ network. However, it decreases the oxygen vacancies of CeO_2_ compared to the reduced 5Ni5Ce/MA catalyst. As a consequent, the reactant cracking, especially CO_2_, on 5Ni5Ce/MA is slower by the metal–oxide pair basic sites and the coke oxidation is driven only by surface oxygen (O*) from the CO_2_ adsorption–dissociation. Moreover, the beneficial pathway of oxygen transfer through the Ni-NiO-CeO_2_ network to the deposited carbon rarely takes place on this catalyst. When the 5Ni5Ce/MA catalyst is treated in the ammonia vapor, the reactant cracking becomes faster and more specific onto the appropriate sites as explained above. Thus, graphitic carbon and a thick carbon filament were found on the treated catalysts. The smaller amount of coke deposited on the treated catalysts represents the higher rate of coke removal. This is attributed to the implementation of oxygen transfer into the Ni-NiO-CeO_2_ network associated with the rehydration property of Ni-NiO-CeO_2_ interactions, indicating more surface hydroxyl (OH)^*^. Ni-NiO-CeO_2_ nanoparticles on 5Ni5Ce(20 h)/MA provide OH* and O* to drive the reaction pathways that transform CH* and C* to CO* (Equations (5)–(7)).

CH* + O* → CHO* + *→ CO* + H*(5)

C* + O* → CO* + *(6)

C* + OH* → CHO* + *→ CO* + H*(7)

## 3. Materials and Methods

### 3.1. Material Preparation

The catalyst support, 10 wt% MgO/Al_2_O_3_ (MA), was synthesized by incipient wetness impregnation of Al_2_O_3_ powder (98%, Sigma Aldrich, St. Louis, MO, USA) with an aqueous solution of Mg(NO_3_)_2_·6H_2_O (98%, Acros Organics, Waltham, MA, USA). The wet powder was stabilized in ambient condition for 12 h before being dried at 50 °C overnight and calcined at 650 °C for 6 h. Afterwards, 5 wt% Ni-5 wt% CeO_2_/MgO-Al_2_O_3_ (5Ni5Ce/MA) was prepared through the conventional co-impregnation method. The mixture of the required portion of the Ni(NO_3_)_2_·6H_2_O (98%, Acros Organics) solution and the Ce(NO_3_)_3_·6H_2_O (98%, Sigma Aldrich) solution was dropped onto the MA support followed by drying at 50 °C overnight and calcination at 650 °C for 6 h.

To generate two treated catalysts with various interactions on the surface, the 5Ni5Ce(xh)/MA (xh = 6 h and 20 h) catalysts were synthesized using an ammonia vapor diffusion impregnation method outlined in our previous work [19]. The wet solid cake of the impregnated 5Ni5Ce/MA catalyst was kept under ammonia vapor diffusion conditions at ambient temperature in a closed chamber for 6 h (5Ni5Ce(6 h)/MA) and for 20 h (5Ni5Ce(20 h)/MA). During the ammonia diffusion, a limited amount of hydroxide ions was intercalated with the nitrate ions of the Ni^2+^ and Ce^3+^ solutions, resulting in the slow growth of the layered double hydroxide nanosheet structure. Hence, the size and dispersion of the layered double hydroxide nanosheets were controlled by diffusion time. The resulting wet powder was dried and calcined using similar conditions to those used for the 5Ni5Ce/MA catalyst. All catalysts were pelletized, ground, and sieved to a particle size range between 355 and 710 μm.

### 3.2. Material Characterization

The surface morphologies of the calcined and spent catalysts were characterized by field emission scanning electron microscopy (FESEM) using the JEOL instrument model JSM-7610F (JEOL Ltd., Welwyn Garden City, UK) with an accelerating voltage of 1.00 kV. Prior to the analyzation, each catalyst sample was prepared by sputter coating with platinum (Pt) using a QUORUM Q150R S (Quorum, East Sussex, UK) apparatus.

The surface area and pore properties of the catalyst were obtained using the N_2_ adsorption–desorption isotherm measured at −196 °C on an Autosorb iQ Station 2 (Quantachrome Instruments, Boynton Beach, FL, USA). Prior to the experiment, the catalyst was outgassed at 350 °C under a N_2_ flow for 3 h. The surface area and the total pore volume were analyzed by multipoint Brunauer–Emmett–Teller (BET) method. The average pore diameter was calculated from the Barett–Joyner–Halenda (BJH) method.

The crystalline phase compositions of the prepared support, calcined catalysts, reduced catalysts, and spent catalysts were identified by X-ray diffraction (XRD, Bruker AXS Model D8 Discover, Billerica, MA, USA) with CuKα radiation at 40 kV and 40 mA and recorded in 2 theta ranges of 10–80° with the step size of 0.02° min^−1^. The average crystallite sizes (D_XRD_) on the fresh catalysts were calculated from the values of full width at half maximum (FWHM) of the intense diffraction peak at 2 theta = 28° for CeO_2_ using Scherrer’s equation (Equation (8)), where K is the particle shape factor (0.94), λ is 1.5418 Å, β is line broadening in radians, and θ is the Bragg angle.

(8) $$D(\mathrm{XRD})=\frac{K\lambda }{\beta \cos\theta }$$

The reducibility of the calcined catalysts was analyzed by hydrogen temperature-programmed reduction (H_2_-TPR) measurements performed on a BELCAT basic system (BEL JAPAN, INC., Osaka, Japan) using a thermal conductivity detector (TCD). A total of 0.050 g of each sample was first degassed with Ar flow at 220 °C for 1.5 h, and cooled down to 40 °C. In the measurement, the sample was reduced in the reducing gas flow (5 vol% H_2_/Ar, 30 mL min^−1^) from 50 °C to 800 °C with a ramping rate of 10 °C min^−1^. The water vapor from the gas stream was removed using a molecular sieve 4A, and the hydrogen consumption was examined by the TCD equipped in the analyzer.

The chemical states of Ni and Ce in the reduced catalysts were investigated by X-ray photoelectron spectroscopy (XPS) measurement, employing a PHI5000 Versa Probe II (ULVAC-PHI, Chigasaki, Japan). Prior to the measurement, catalysts were reduced ex situ at 600 °C for 3 h under pure H_2_. The monochromatic AlKα X-ray (1486.6 eV) was utilized as an excitation source. The C 1s BE at 285.0 eV was used as a reference for calibration. The high-resolution XPS spectra were collected with an energy step of 0.1 eV and pass energy of 46.95 eV. After measurement, XPS spectra were analyzed using the CasaXPS software (version 2.3.26).

The dispersion of metal active sites was investigated via H_2_ temperature-programmed desorption (H_2_-TPD) measured on the BELCAT basic system. Firstly, the catalyst sample (0.050 g) was pre-treated in Ar flow (30 mL min^−1^) at 220 °C for 1 h, reduced in situ with pure H_2_ flow (30 mL min^−1^) at 600 °C for 1.5 h, and cooled to 120 °C in Ar flow. After that, the H_2_ adsorption on the reduced catalyst was carried out in pure H_2_ flow at 120 °C for 1h. The physiosorbed H_2_ was purged with Ar flow for 1 h, and the sample was cooled to 50 °C. Finally, the H_2_-TPD measurement was performed from 50 °C to 850 °C at the heating rate of 10 °C min^−1^ in Ar flow.

The surface alkalinity and basic strength distribution of the reduced catalysts were evaluated by CO_2_ temperature-programmed desorption (CO_2_-TPD) conducted on the same BELCAT apparatus. Prior to the experiment, the catalyst sample was pre-treated in He flow (30 mL min^−1^) at 220 °C for 1 h, and reduced in situ with pure H_2_ flow (30 mL min^−1^) at 600 °C for 1.5 h. The sample was thereafter cooled to 40 °C in He flow, and an isothermal CO_2_ adsorption was subsequently introduced to the catalyst surface at 40 °C for 1 h. The unadsorbed CO_2_ was removed by flushing with He flow for 1 h. Then, the CO_2_ desorption was measured with a heating rate of 10 °C min^−1^ to 800 °C in He flow.

The total amount of oxygen mobility and the distribution of the mobile species were determined via temperature-programmed desorption of oxygen (O_2_-TPD) on the same BELCAT instrument (Microtrac, York, PA, USA). Before the O_2_ adsorption step, the catalyst sample was pre-treated and pre-reduced in situ under the same conditions as H_2_-TPD. The sample was cooled to 200 °C in Ar flow. Afterward, O_2_ adsorption on the reduced catalyst was carried out in pure O_2_ flow at 200 °C for 1.5 h and the physiosorbed O_2_ was removed with Ar flow. Then, the desorption was monitored from 50 °C to 800 °C at a heating rate of 10 °C min^−1^ in Ar flow.

The total carbon deposition and carbon type distribution on the spent catalysts after the CRM reaction test were elucidated by temperature-programmed oxidation (TPO) using the BELCAT basic system. A total of 0.050 g of each spent catalyst was cleaned in Ar flow (30 mL min^−1^) at 220 °C for 2 h, followed by cooling to 40 °C before the TPO measurement. Then, the cleaned sample was oxidized in a 5 vol.% O_2_/Ar flow (30 mL min^−1^) with a ramping rate of 10 °C min^−1^ from 50 °C to 800 °C. The CO_2_ product that evolved from the catalyst surface was removed by a 4A molecular sieve and the oxygen consumption was determined by the TCD equipped in the analyzer.

### 3.3. Catalytic Performance Tests

The CRM was tested in a tubular reactor at 600 °C under atmospheric pressure for 16 h. Prior to each test, a 350 mg catalyst sample was reduced in situ under H_2_ flow (30 mL min^−1^) at 600 °C for 3 h. Then, the reaction feedstock of a CH_4_:CO_2_:N_2_ mixture with a molar ratio of 3:5:4 at the total flow rate of 60 mL min^−1^ was introduced into the reactor. The composition of the outlet stream was analyzed using an on-line gas chromatograph (Agilent GC7890A, Santa Clara, CA, USA) equipped with a TCD. The reactant conversions (Equations (9) and (10)), product yields (Equations (11) and (12)), and H_2_/CO ratio (Equation (13)) were calculated by the equations below.

(9) $$\%CH_{4}\;\mathrm{conversion}=\frac{\Delta \mathrm{Flow}\;\mathrm{rate}\;\mathrm{of}\;CH_{4}}{\mathrm{Flow}\;\mathrm{rate}\;\mathrm{of}\;CH_{4,\mathrm{in}}}\;\times \;100$$

(10) $$\%CO_{2}\;\mathrm{conversion}=\frac{\Delta \mathrm{Flow}\;\mathrm{rate}\;\mathrm{of}\;CO_{2,\mathrm{in}}}{\mathrm{Flow}\;\mathrm{rate}\;\mathrm{of}\;CO_{2,\mathrm{in}}}\;\times \;100$$

(11) $$\%H_{2}\;\mathrm{yield}=\frac{\mathrm{Flow}\;\mathrm{rate}\;\mathrm{of}H_{2,\mathrm{out}\;}}{2(\Delta \mathrm{Flow}\;\mathrm{rate}\;\mathrm{of}\;CH_{4})}\;\times \;100$$

(12) $$\%\mathrm{CO}\;\mathrm{yield}\;=\frac{\mathrm{Flow}\;\mathrm{rate}\;\mathrm{of}\;{\mathrm{CO}}_{,\mathrm{out}\;}}{\Delta \mathrm{Flow}\;\mathrm{rate}\;\mathrm{of}\;CH_{4}+\Delta \mathrm{Flow}\;\mathrm{rate}\;\mathrm{of}\;CO_{2}}\;\times \;100$$

(13) $$\frac{H_{2\;}}{\mathrm{CO}}\;\mathrm{ratio}=\frac{\mathrm{Flow}\;\mathrm{rate}\;\mathrm{of}\;H_{2,\;\mathrm{out}}}{\mathrm{Flow}\;\mathrm{rate}\;\mathrm{of}\;CO_{,\mathrm{out}}}$$

## 4. Conclusions

This study discloses the effect of the growth of Ni-NiO-CeO_2_ nanoparticles on 5 wt% Ni-5 wt% CeO_2_/MA (treated) catalyst employing impregnation-assisted ammonia vapor diffusion for 6 h and 20 h. Compared to the untreated 5 wt%Ni-5 wt%CeO_2_/MA, the growth of Ni-NiO-CeO_2_ nanoparticles constructed a Ni-NiO-CeO_2_ network on the MA support with electronic interaction. The short duration of ammonia vapor diffusion initiates the electronic interaction of Ni-NiO-CeO_2_, interrupts strong metal–support interaction, creates a high dispersion of small Ni-NiO-CeO_2_ nanoparticles, and increases surface hydroxyl basic sites. A stronger interaction in the Ni-NiO-CeO_2_ network is developed over the ammonia diffusion time. Ni metal and the surface hydroxyl sites on Ni-NiO-CeO_2_ nanostructures with lower metal–support interaction raise catalytic activities toward involving a drastic reactant dissociation. Although the hard removal types of coke are formed on treated catalysts, the rate of coke oxidation is dramatically driven by mobile O* and OH* in strong Ni-NiO-CeO_2_ interactions.

## Figures and Tables

**Table 1:** Bulk and surface properties of support and all calcined catalysts.

Sample	Surface Area (m^2^ g^−1^)	Total Pore Volume (cm^3^ g^−1^)	Average Pore Diameter (nm) ^a^	Crystallite Size of CeO_2_ (nm) ^b^
MgO-Al_2_O_3_	151	0.52	8.0	-
5Ni5Ce/MA	108	0.51	10.8	6.7
5Ni5Ce(6 h)/MA	101	0.44	9.3	5.4
5Ni5Ce(20 h)/MA	101	0.43	10.8	7.2

^a^ Average pore diameter determined by BJH model. ^b^ Crystallite size of CeO_2_ calculated by Scherrer’s equation.

**Table 2:** Chemical state analysis of Ni 2p, Ce 3d, and O 1s from XPS spectra.

Samples	$\frac{\left[{\mathrm{Ni}}^{0}\right]}{\left[{\mathrm{Ni}}^{0}\right]+\left[{\mathrm{Ni}}^{2+}\right]}$	$\frac{\left[{\mathrm{Ce}}^{3+}\right]}{\left[{\mathrm{Ce}}^{3+}\right]+\left[{\mathrm{Ce}}^{4+}\right]}$	O_sur_/O_latt_
5Ni5Ce/MA	0.45	31.30	0.88
5Ni5Ce(6 h)/MA	0.22	28.30	0.47
5Ni5Ce(20 h)/MA	0.29	25.61	0.52

**Table 3:** The amount of H_2_ desorption on all reduced catalysts.

Samples	Amount of H_2_ Desorption (mmol g^−1^)		
	Weak 100–180 °C	Medium–Strong 180–750 °C	$\frac{\mathrm{Weak}}{(\mathrm{Medium}+\mathrm{Strong})}$
5Ni5Ce/MA	0.021	0.167	0.126
5Ni5Ce(6 h)/MA	0.032	0.166	0.191
5Ni5Ce(20 h)/MA	0.066	0.123	0.537

**Table 4:** The amount of CO_2_ desorption on all reduced catalysts.

Samples	Amount of CO_2_ Desorption (mmol g^−1^)			
	Weak 50–150 °C	Moderate 150–400 °C	Strong 400–800 °C	Total Basicity
5Ni5Ce/MA	0.872	1.432	0.643	2.947
5Ni5Ce(6 h)/MA	0.918	1.396	0.688	3.002
5Ni5Ce(20 h)/MA	0.964	0.973	0.501	2.438

**Table 5:** The amount of O_2_ desorption on all reduced catalysts.

Samples	Amount of O_2_ Desorption (mmol g^−1^)		
	Surface Oxygen (<150 °C)	Lattice Oxygen (>150 °C)	Total O_2_ Desorption
5Ni5Ce/MA	0.353	1.236	1.589
5Ni5Ce(6 h)/MA	0.259	1.604	1.863
5Ni5Ce(20 h)/MA	0.366	1.051	1.417

**Table 6:** The O_2_ consumption for carbon oxidation on all spent catalysts.

Samples	O_2_ Consumption (mmol g^−1^)			
	Amorphous Carbon (<320 °C)	Graphitic Carbon (320–550 °C)	Filamentous Carbon (>550 °C)	Total O_2_ Consumption
5Ni5Ce/MA	0.87	13.53	2.62	17.02
5Ni5Ce(6 h)/MA	0.94	6.11	4.81	11.86
5Ni5Ce(20 h)/MA	0.68	5.21	2.52	8.4

## Data Availability

Data are contained within the article.
